# Joint Optimization of Packet Survivability and Aerodynamic Energy for Dynamic UAV Activation in VANETs via Deep Q-Networks

**DOI:** 10.3390/s26165190

**Published:** 2026-08-17

**Authors:** Prangya Priyadarshini, Arun Kumar

**Affiliations:** Department of Computer Science and Engineering, National Institute of Technology, Rourkela 769008, India; 522cs3005@nitrkl.ac.in

**Keywords:** deep reinforcement learning, UAV-assisted VANETs, network survivability, three-component energy model, 6G communications, resource provisioning

## Abstract

UAV-assisted VANETs are a key component of the 6G vision, yet their practical deployment is hindered by the fundamental conflict between network Quality of Service (QoS) and the high aerodynamic power required for rotary-wing flight. This paper proposes SAVIOR (Survivable Aerial-Vehicular Intelligent Optimization and Routing), a Deep Reinforcement Learning (DRL) framework that jointly optimizes multi-UAV activation and packet routing. Unlike existing approaches that rely on oversimplified linear energy models, SAVIOR integrates a rigorous three-component aerodynamic power model and introduces an M/M/1 queuing-based Survivability Score (*S*-score) to explicitly quantify packet delivery before Time-to-Live (TTL) expiration. Through a high-fidelity co-simulation using SUMO and Python-TraCI, the SAVIOR agent is able to handle stress-test situations where network demand is higher than capacity (ρ>1.0). A comparative analysis shows that SAVIOR is Pareto-optimal, with a total reward that is 65% higher than that of a static energy-saving policy and a survivability that is 24% higher. Crucially, compared to a performance-maximizing greedy policy, SAVIOR maintains comparable safety-critical QoS while reducing total energy consumption by 19.8%, thereby preventing premature battery depletion and mitigating co-channel interference.

## 1. Introduction

The vision of modern smart cities is inextricably linked to the development of Intelligent Transportation Systems (ITS), where Vehicular Ad hoc Networks (VANETs) serve as the fundamental communication backbone [1]. VANETs enable a seamless exchange of data through Vehicle-to-Vehicle (V2V) and Vehicle-to-Infrastructure (V2I) paradigms, facilitating a wide array of applications ranging from safety-critical collision avoidance and cooperative adaptive cruise control to high-bandwidth passenger infotainment services [2]. Unlike traditional mobile networks, VANETs are characterized by high node mobility, rapid topology changes, and a diverse set of Quality of Service (QoS) requirements. These intrinsic characteristics introduce formidable optimization challenges, particularly concerning reliable data dissemination and resource management under varying traffic densities.

Despite significant advancements in Dedicated Short-Range Communications (DSRC) and Cellular-V2X (C-V2X) standards [3], conventional VANET architectures, which rely predominantly on static Roadside Units (RSUs), continue to face fundamental limitations. In dense urban environments, signals are frequently obstructed by buildings, leading to Non-Line-of-Sight (NLoS) conditions and intermittent connectivity. Moreover, the deployment of fixed RSUs is often economically unfeasible in sparse rural areas or physically impossible in disaster-stricken regions. Furthermore, the massive control overhead required to maintain routing paths in a high-mobility grid often leads to network saturation and excessive latency [4].

**Figure 1:**
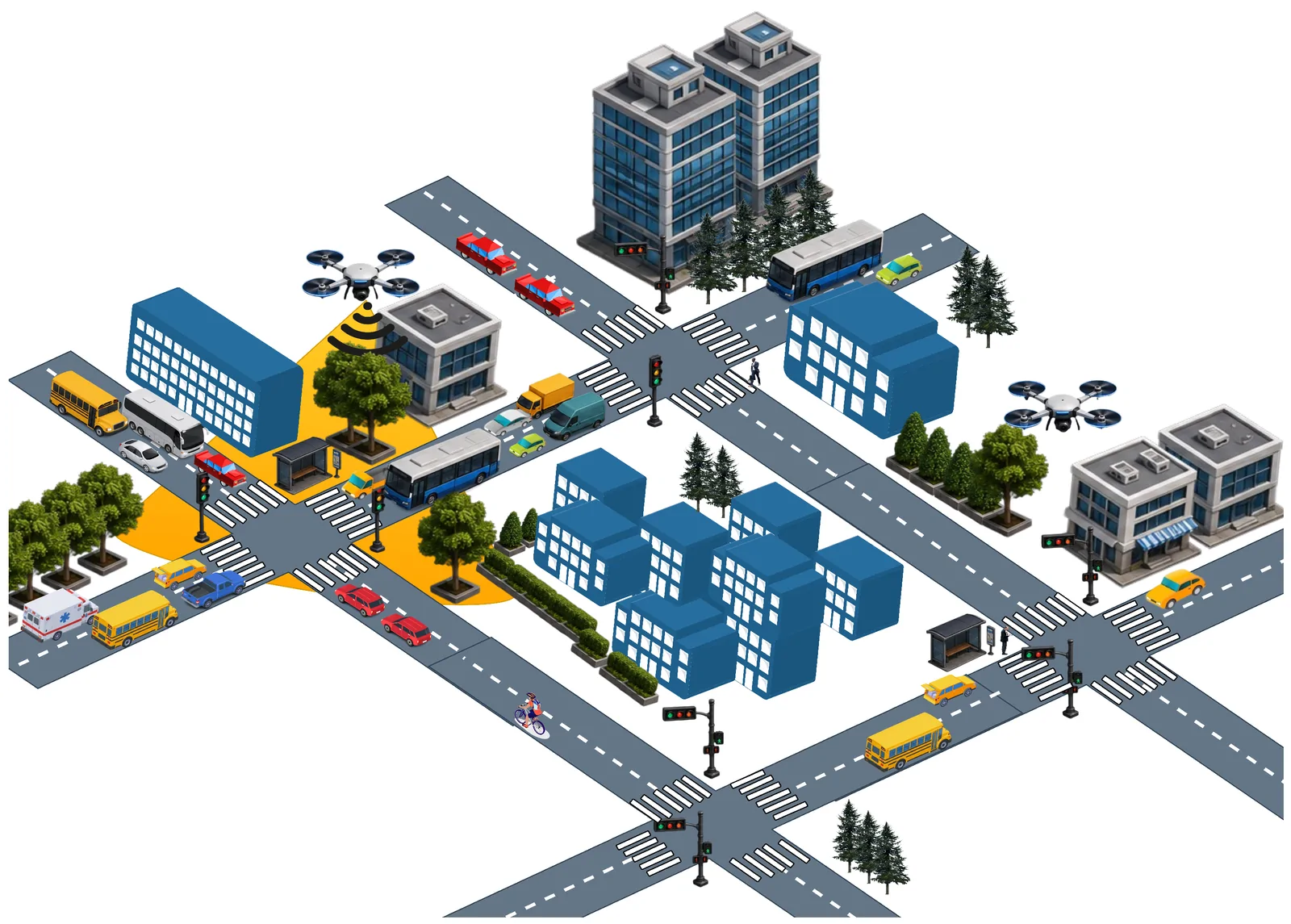
System scenario of the SAVIOR framework.

To address these challenges and realize the sixth-generation (6G) vision of space–air–ground integrated networks, Unmanned Aerial Vehicles (UAVs) have gained prominence as aerial base stations and mobile relays within Non-Terrestrial Networks (NTNs). Positioned at optimal altitudes (typically 50–150 m), UAVs establish high-probability Line-of-Sight (LoS) communication links with ground vehicles, significantly mitigating the shadowing effects observed with terrestrial RSUs [5]. The three-dimensional mobility of UAVs allows for dynamic coverage adaptation, where aerial nodes can be repositioned to cover traffic hotspots or bridge network partitions as shown in Figure 1. Recent studies indicate that UAV-assisted VANETs can meet 6G Ultra-Reliable Low Latency Communication (URLLC) requirements by substantially improving the Packet Delivery Ratio (PDR) and lowering end-to-end latency [6].

However, the integration of UAVs into sustainable 6G ecosystems introduces a complex multi-objective optimization problem driven by severe energy constraints. Unlike ground vehicles with near-unlimited fuel capacity, rotary-wing UAVs rely on lithium-polymer batteries with limited operational durations, typically spanning 10 to 30 min [7]. In these platforms, propulsion power is the dominant energy sink, accounting for 85–95% of the total energy budget [8]. Consequently, the fundamental challenge lies in the conflicting nature of energy consumption and QoS provisioning. Activating a larger fleet of UAVs improves network capacity and reduces queuing delays, thereby enhancing packet survivability, defined as the probability of delivery before a Time-to-Live (TTL) expiration. However, this over-provisioning leads to a proportional increase in aerodynamic energy expenditure, violating the “Green Communications” Key Performance Indicators (KPIs) of 6G systems [9,10].

Optimizing this trade-off is mathematically non-trivial due to the non-linear relationship between UAV flight dynamics and network load factors. The existing literature generally addresses these challenges through three paradigms. First, model-based methods employ game theory or convex optimization to jointly optimize trajectories and resource allocation [11]. While rigorous, these methods often require a priori knowledge of traffic patterns and struggle to adapt to the stochastic, real-time dynamics of vehicular movement. Second, heuristic algorithms, such as greedy activation policies, provide low computational complexity but lack optimality guarantees, often leading to energy-wasteful “always-on” configurations [12]. Third, Deep Reinforcement Learning (DRL) has emerged as a powerful tool for AI-native 6G networking, offering the flexibility to learn control policies from environmental interaction. Nevertheless, many current DRL solutions focus exclusively on single-UAV trajectory design or overlook the complex, three-component energy dynamics (blade, induced, and parasite power) involved in multi-UAV activation decisions [13].

The key contributions of this work are as follows:

- Multi-Objective Reinforcement Learning Formulation: The UAV activation problem is formulated as a multi-objective Markov Decision Process (MDP) that balances survivability maximization against energy minimization. Through a carefully designed reward function, the proposed formulation enables the learning agent to discover Pareto-optimal operating points along the energy–QoS trade-off frontier.
- Dynamic UAV Activation Policy: A Deep Q-Network (DQN) agent is created that learns how to dynamically turn on a group of available UAVs based on the current state of the network, such as vehicle density, load factors, and battery levels. This separate action space makes it easy to manage energy with great detail, even when traffic changes.
- Realistic Integration with SUMO Traffic Simulator: This approach is implemented using the Simulation of Urban Mobility (SUMO) framework with realistic vehicle mobility patterns and IEEE 802.11g communication modeling. To rigorously evaluate the efficacy of the SAVIOR agent, its performance is compared against three specialized operational strategies: a Static (energy-conservative) policy, a Greedy (performance-maximizing) policy, and a Random activation baseline.

While the SAVIOR framework demonstrates significant performance and energy gains, its current formulation is subject to certain limitations. Primarily, the system utilizes a centralized DRL controller, which may encounter scalability and signaling overhead constraints in ultra-dense, large-scale vehicular deployments. Additionally, the study focuses on the dynamic activation logic for UAVs at a fixed altitude and does not yet incorporate continuous three-dimensional trajectory optimization or autonomous collision avoidance between aerial nodes. Furthermore, the communication model employs a macroscopic interference approximation rather than a microscopic physical-layer Signal-to-Interference-plus-Noise Ratio (SINR) analysis. These factors provide a clear roadmap for our future research, which will explore decentralized Multi-Agent Reinforcement Learning (MARL) and joint trajectory-activation control to address these constraints.

The subsequent sections of the paper are as follows. Section 2 delineates the literature survey. The design of the proposed SAVIOR algorithm is shown in Section 3. Section 4 depicts the analysis of the algorithms, followed by simulation results. Finally, this research work is concluded in Section 6. To enhance readability, the notations used in the work are listed in Abbreviations.

## 2. Related Work

The improvement of UAV-assisted vehicular networks has attracted significant research attention, focusing mainly on two specific challenges: energy-efficient routing protocols and physical trajectory planning. The current literature can be generally classified into heuristic-based routing, Reinforcement Learning (RL) for packet forwarding, and trajectory optimization for energy efficiency.

### 2.1. Energy-Aware Routing in UAV Networks

Routing in highly dynamic Flying Ad Hoc Networks (FANETs) and UAV-assisted VANETs necessitates protocols capable of accommodating frequent alterations in network topology. Initial research efforts have leveraged swarm intelligence and filtering heuristics to manage these dynamics. For instance, the ECRIA-UAV framework [14] utilizes an Improved Ant Colony Optimization (IACO) algorithm to enhance route discovery and maximize the Packet Delivery Ratio (PDR). While this approach effectively reduces routing overhead and information delay, it relies on heuristic path selection and lacks a rigorous three-component aerodynamic power model for the UAV platforms. Similarly, the LFEAR scheme [15] employs local filtering and movement similarity metrics to mitigate broadcast storms and ensure path stability. However, this heuristic strategy focuses primarily on route discovery control and neglects the dominant cost of aerodynamic propulsion and dynamic load-adaptive activation logic.

To address the stochastic nature of vehicular traffic, learning-based methodologies have gained prominence due to their adaptive nature. The Q-LBR protocol [16] employs tabular Q-learning and queue status monitoring to achieve effective load balancing and bottleneck mitigation. Although it significantly improves network utilization, its optimization scope is restricted to network-layer congestion, neglecting the discrete UAV activation logic and non-linear energy expenditure. More recent advancements utilize Deep Reinforcement Learning (DRL) to manage high-dimensional state spaces. The fast routing protocol proposed in [17] utilizes deep neural networks to minimize latency violations and state quantization errors in large-scale deployments. Furthermore, the MPEAOLSR protocol [18] optimizes multi-point relay selection to minimize control overhead in infrastructure-less environments.

While these RL-based schemes demonstrate superior adaptability, a fundamental limitation across the existing routing literature is the reliance on simplified energy abstractions. These studies usually consider energy drain as a linear function of time or distance, overlooking the non-linear aerodynamic power curves inherent to rotary-wing flight. Specifically, they fail to account for the high power cost of hovering compared to optimal cruise velocities, a physical reality that the SAVIOR framework integrates directly into the routing decision process to ensure sustainable and survivable communication.

### 2.2. UAV Trajectory and Physical Energy Optimization

A separate body of literature focuses on the physical movement of UAVs, where propulsion energy constitutes the primary energy sink. Optimizing this layer is a complex control problem due to the non-linear nature of rotary-wing aerodynamics. To achieve high algorithmic rigor, some studies employ Mixed Integer Programming (MIP) [19] to optimize battery-constrained fleet scheduling and battery-swapping logistics for urban surveillance. While MIP-based methods provide provable optimality for deterministic tasks, the inherent computational latency of exact solvers limits their applicability to the highly stochastic, real-time traffic patterns of VANETs.

Consequently, research has shifted toward more agile, scenario-specific trajectory designs. For instance, a “fly-circle-communicate” algorithm [20] was introduced to minimize mission time in IoT networks using a non-linear model that accounts for motor dynamics. Similarly, AoI-and-energy-aware schemes [21] utilize successive convex approximation to minimize completion time while ensuring data freshness. Under harsh environmental conditions, trajectory designs have been re-evaluated to account for weakened battery discharge capacities [22]. While these methods effectively optimize physical endurance for sequential visitation tasks, they typically assume static or expected data demands, making them less suitable for the overlapping, high-frequency data bursts characteristic of dense vehicular traffic. In emergency contexts, dual cost-aware multi-armed bandit algorithms [23] have been used to maximize user coverage under uncertainty; however, these focus on visitation metrics rather than packet-level survivability.

More sophisticated approaches incorporate 3D positioning and multi-agent coordination. The MADDPG algorithm [24] has been successfully utilized for 3D trajectory optimization and task offloading in Multi-access Edge Computing (MEC) systems, ensuring fairness and energy efficiency among aerial nodes. While these studies utilize precise energy models that account for hovering and vertical motion, a recurring limitation is the decoupling of the physical layer from the networking layer. By treating data traffic as fixed demand points rather than dynamic packet flows subject to queuing delays and Time-to-Live (TTL) constraints, a trajectory optimized purely for flight energy may unintentionally lead to network congestion or catastrophic packet expiration. The SAVIOR framework addresses this gap by jointly optimizing aerodynamic power and queuing-theoretic survivability.

### 2.3. Deep Reinforcement Learning (DRL) in UAV-VANETs

Recent advancements have leveraged DRL to handle the high-dimensional state spaces inherent to aerial-vehicular networks. Within this domain, DRL has been successfully applied to enhance connectivity and routing reachability. For instance, the MASAC-based protocol in [25] utilizes soft actor–critic logic to optimize trajectory planning, significantly improving the PDR in sparse topologies. While effective for data dissemination, its primary focus remains on routing efficiency, lacking a rigorous aerodynamic power model to account for the substantial propulsion overhead of hovering during relay tasks. To address hardware constraints, a “Tiny RL” framework [26] was proposed using MADDPG and network pruning to minimize onboard computational overhead and memory usage. Although this provides a lightweight solution for embedded systems, the model prioritizes hardware-level optimizations over the non-linear aerodynamic power dynamics and queuing-theoretic survivability of the network.

To mitigate the limitations of centralized control, decentralized and collaborative DRL architectures have been explored. A CMAD-DDQN approach is developed [27] that maximizes system energy efficiency by sharing telemetry among neighboring UAV agents. While this collaborative framework improves coordination and interference handling in dynamic environments, it targets general coverage objectives rather than the specific delay and TTL-constrained survivability requirements of VANETs. Furthermore, research has shifted toward hybrid control strategies to balance transmission costs and physical stability. A DQN-based strategy [28] was proposed to optimize the trade-off between transmission energy and tracking accuracy; however, it focuses on signal-level efficiency and neglects the dominant propulsion costs. Similarly, the hybrid DRL-ANMPC framework in [29] provides high-fidelity trajectory tracking robust to physical disturbances, but this control-centric perspective overlooks the communication-layer load dynamics and packet expiration deadlines.

A fundamental drawback common to these existing works is the oversimplification of the UAV power profile. While these studies demonstrate the feasibility of onboard AI, they often treat energy as a linear function of time or distance, ignoring the critical aerodynamic reality that hovering energy consumes significantly more power than forward flight due to the lack of translational lift. By neglecting this physical constraint, existing DRL models may inadvertently incentivize UAVs to maintain stationary positions to maximize connectivity, thereby draining batteries at the maximum possible rate.

Table 1 provides a comparative summary of existing UAV-VANET frameworks and highlights the critical research gaps addressed by the SAVIOR approach.

To address these limitations, this work proposes a foresight-guided DRL framework that integrates a realistic Three-Component Energy Model (accounting for blade, induced, and parasite power) directly into the routing decision process. Unlike prior works, the proposed approach jointly optimizes the discrete activation of UAVs and packet routing to maximize a novel survivability score (S-score) while minimizing total system energy.

## 3. System Model and Methodology

This section details the proposed **SAVIOR** (Survivable Aerial-Vehicular Intelligent Optimization and Routing) framework. Firstly, the physical system model is defined, encompassing the dynamic network architecture, channel characteristics, and the non-linear rotary-wing energy model. Subsequently, the UAV activation problem is formalized as a multi-objective Markov Decision Process (MDP) and the DRL agent is designed to solve it.

### 3.1. Network Architecture

A UAV-assisted VANET is deployed over a discretized urban environment. The system comprises a set of *N* rotary-wing UAVs, $U=\{u_{1},\ldots ,u_{N}\}$, acting as aerial base stations for a time-varying set of ground vehicles V(t). Each UAV operates at a fixed altitude *h* to ensure LoS coverage. Vehicular traffic follows dynamic mobility patterns, and a centralized controller determines the number of active UAVs K(t)∈{1,…,N} at discrete decision intervals to optimize network performance.

### 3.2. Communication and Interference

The Air-to-Ground (A2G) channel is modeled using a log-distance path loss model with shadowing. To capture the degradation of link quality in dense traffic, an interference-aware effective capacity model is introduced. For a UAV $u_{i}$, the effective channel capacity $C_{eff,i}\left(t\right)$ is given by:

(1) $$C_{eff,i}\left(t\right)=\frac{C_{nom}}{\eta_{i}\left(t\right)},\;\mathrm{where}\;\eta_{i}\left(t\right)=1+\delta \cdot I_{i}\left(t\right)$$

where $C_{nom}$ represents the nominal link capacity, $I_{i}\left(t\right)$ denotes the number of active interfering links in the vicinity, and δ is the interference penalty coefficient.

The scaling factor $\eta_{i}\left(t\right)$ is adopted as a macroscopic phenomenological approximation to capture the aggregate impact of co-channel interference and MAC-layer contention. In IEEE 802.11-based vehicular networks, especially in dense urban environments, the effective capacity is predominantly constrained by the CSMA/CA mechanism, where a higher density of interfering transmitters $I_{i}\left(t\right)$ increases the likelihood of Clear Channel Assessment (CCA) busy events and exponential backoff. Rather than modeling microscopic physical-layer SINR dynamics, $\eta_{i}\left(t\right)$ serves as a computationally efficient surrogate for DRL training that approximates the reduction in transmission airtime and effective saturation throughput as local node density increases. This formulation preserves mathematical tractability while providing a physically consistent congestion-aware penalty signal for the SAVIOR agent.

### 3.3. Traffic and Queuing Dynamics

Traffic is modeled as a Poisson process where vehicles generate safety-critical packets of size *L* with an arrival rate $\lambda_{v}$. The buffering process at each UAV is modeled as an M/M/1 queue. The utilization factor (load) $\rho_{i}\left(t\right)$ is defined as the ratio of aggregate arrival rate to service rate:

(2) $$\rho_{i}\left(t\right)=\frac{\lambda_{i}\left(t\right)}{\mu_{i}\left(t\right)}=\frac{M_{i}\left(t\right)\cdot \lambda_{v}\cdot L}{K\left(t\right)\cdot C_{eff,i}\left(t\right)}$$

While steady-state M/M/1 queuing analysis requires $\rho_{i}\left(t\right)<1$ for stability, the SAVIOR framework is specifically evaluated under transient high-load conditions in which demand may temporarily exceed service capacity ($\rho_{i}\left(t\right)\geq 1.0$). In this work, the queuing delay term $W_{q}\left(t\right)$ is utilized as a finite-horizon congestion indicator for reward shaping rather than as a strict steady-state predictor. For operational regimes where $\rho_{i}\left(t\right)<0.95$, $W_{q}\left(t\right)$ is computed using the standard M/M/1 expression. However, when $\rho_{i}\left(t\right)\geq 0.95$, the delay is clipped at a threshold of 0.9τ:

(3) $$W_{q}\left(t\right)=0.9\tau$$

This clipping provides a conservative margin before the hard Time-to-Live (TTL) deadline τ, ensuring that packets approaching expiration are correctly penalized by the RL agent. To capture the physical reality of buffer overflows and deadline misses during these stress-test periods, we additionally compute and report the empirical packet loss ratio, defined as the fraction of dropped or TTL-expired packets over the total number of generated packets during saturated intervals.

For mathematical tractability, the following assumptions are adopted: (i) packet arrivals at each vehicle follow a Poisson process, (ii) service times at a UAV are exponentially distributed, (iii) vehicles are uniformly associated among the active UAVs, and (iv) interference affects capacity through an aggregate penalty factor derived from the transmitter count $I_{i}\left(t\right)$. These assumptions facilitate an analytically tractable survivability metric while capturing the dominant congestion and energy trends in dense urban VANETs.

### 3.4. Energy Consumption Model

A significant contribution of this work is the incorporation of a precise aerodynamic power model [30]. The instantaneous power P(V) of a rotary-wing UAV is computed as the aggregate of three components—Blade Profile, Induced Power, and Parasite Power—in contrast to simplified linear models.

(4) $$P\left(V\right)=\underset{\mathrm{Blade}\;\mathrm{Profile}}{\underset{︸}{P_{b}\left(1+\frac{3V^{2}}{U_{tip}^{2}}\right)}}+\underset{\mathrm{Induced}}{\underset{︸}{P_{i}\sqrt{\sqrt{1+\frac{V^{4}}{4v_{0}^{4}}}-\frac{V^{2}}{2v_{0}^{2}}}}}+\underset{\mathrm{Parasite}}{\underset{︸}{\frac{1}{2}d_{0}\rho_{air}sAV^{3}}}$$

where *V* denotes the UAV’s forward velocity. The physical significance of these components is as follows:

- Blade Profile Power ($P_{b}$): This represents the energy dissipated to overcome the viscous drag on the rotating rotor blades. This cost exists even at hover and increases quadratically with *V* due to higher relative airspeed over the advancing blades.
- Induced Power ($P_{i}$): This accounts for the power required to generate lift (thrust) by accelerating air downwards. This component is highest at hover but decreases as *V* increases. This phenomenon, known as translational lift, occurs because the rotor system becomes more efficient as it moves into undisturbed air, reducing the power required to maintain altitude.
- Parasite Power ($P_{p}$): It shows the aerodynamic drag that acts on the UAV fuselage (the parts that do not lift) as it moves through the air. This component scales cubically with velocity ($V^{3}$) and becomes the dominant energy sink at high speeds, imposing a significant penalty on rapid repositioning maneuvers.

The total energy consumption E(t) over the decision interval Δt for K(t) active UAVs is calculated as:

(5) $$E\left(t\right)=K\left(t\right)\cdot \left[P\left(V\right)+P_{comm}\right]\cdot \Delta t$$

where P(V) represents the dynamic propulsion power defined in (4) and $P_{comm}$ accounts for the power consumption of the onboard communication circuitry. While $P_{comm}$ is relatively small compared to the propulsion power, its inclusion ensures a high-fidelity representation of the total battery drain during data transmission tasks.

This model makes the optimization landscape more complicated. For example, hovering (V=0) avoids parasite drag but costs the most power. On the other hand, flying at the best cruise speed can use less energy than staying still.

To ensure the physical fidelity of energy modeling during repositioning, the framework distinguishes between steady-state operation and activation transitions. When the agent maintains its current deployment configuration, the UAVs operate in hovering mode, and the velocity parameter in Equation (4) is set to V=0 m/s, where induced power constitutes the primary energy sink. Conversely, if the selected action $a_{t}$ necessitates a modification in the active fleet size or a shift in the target service region, the velocity is set to a transit constant $V=V_{transit}$, where $V_{transit}=15$ m/s for the duration of that decision interval. This mechanism explicitly incorporates the high aerodynamic overhead of acceleration and parasite drag into the reward signal, effectively penalizing high-frequency activation switching unless such transitions yield substantial gains in network survivability.

### 3.5. Optimization Problem Formulation

Based on the system model, the SAVIOR framework is defined as a multi-objective constrained optimization problem. The goal is to dynamically provision aerial resources to maximize network utility over an operational mission horizon *T*.

#### 3.5.1. Decision Variables

The optimization is driven by two primary decision variables at each decision interval *t*. First one is Activation State ($x_{i}\left(t\right)$). This is a binary variable where $x_{i}\left(t\right)\in \left\{0,1\right\}$ represents the operational state (Off/On) of UAV $u_{i}\in U$. The total active fleet size is given by $K\left(t\right)=\sum_{i=1}^{N}x_{i}\left(t\right)$. The second one is Region Assignment ($\ell_{i}\left(t\right)$), which is a categorical variable $\ell_{i}\left(t\right)\in L$ assigning an active UAV $u_{i}$ to one of ten spatial centroids defined within the grid topology.

#### 3.5.2. Objective Function

The objective is to maximize the cumulative system utility *J*, which balances network reliability against energy expenditure. The problem is formulated as:

(6) $$\max_{\left\{x_{i},\ell_{i}\right\}}\sum_{t=0}^{T}\left(\kappa_{S}\cdot \bar{S}\left(t\right)-\kappa_{E}\cdot E\left(t\right)\right)$$

where $\bar{S}\left(t\right)$ is the aggregate survivability score and E(t) is the total energy consumption (J). The coefficients $\kappa_{S}$ and $\kappa_{E}$ allow for tuning the trade-off between strict QoS adherence and green communication requirements.

#### 3.5.3. Operational Constraints

The decision space is restricted by the following physical and environmental constraints:

- Resource Bounds: 1≤K(t)≤N,∀t. This ensures at least one UAV provides baseline connectivity while respecting the physical limitations of the UAV fleet.
- Flight Safety: $d\left(u_{i},u_{j}\right)\geq d_{sep},\;\forall i\neq j$. Active UAVs are assigned to distinct spatial centroids to maintain a minimum safety buffer, thereby inherently avoiding mid-air collisions.
- Congestion Handling: Unlike strict steady-state models, overload states ($\rho_{i}\left(t\right)\geq 1$) are treated as feasible but highly undesirable. These states are managed through the reward design rather than being excluded from the decision space, allowing the framework to learn recovery policies under high-traffic stress.

By formalizing the problem in the above way, the subsequent SAVIOR methodology serves as a stochastic solver for this optimization model, where the DQN agent learns to navigate these constraints to achieve a desirable survivability–energy trade-off.

### 3.6. The SAVIOR Methodology

The SAVIOR agent is the most important part of the proposed framework. It makes decisions about when to activate the UAVs in real time to balance the conflicting goals of maximizing survivability and minimizing energy.

#### 3.6.1. Survivability Score (S-Score)

To quantify QoS for time-critical applications, the Survivability Score is introduced. This metric goes beyond simple delay by explicitly accounting for the TTL constraint τ. The score $S_{i}\left(t\right)$ is defined as:

(7) $$S_{i}\left(t\right)=C\left(\frac{\tau -W_{q}\left(t\right)}{ETD}\right)$$

C(x)=min(10,max(0,x)) is chosen to bound the score into [0,10]. A value $S_{i}\left(t\right)\geq 1$ indicates that the expected queuing delay is within the end-to-end budget (acceptable QoS), while $S_{i}\left(t\right)=0$ corresponds to packets that are effectively expired.

#### 3.6.2. MDP Formulation

The control problem is represented as a Markov Decision Process (MDP) defined by the tuple (S,A,P,R,γ):

State Space (S): The state vector $s_{t}$ represents the network’s current state, including the amount of traffic, the number of active resources, the current QoS, the load on the network, the amount of interference, and the amount of energy being used.

Action Space (A): The action $a_{t}$ is chosen from a limited set, and then used to find the best number of active UAVs K(t+1). The agent can learn small switching thresholds with this fine-grained action space.

Reward Function (R): The reward function $r_{t}$ pushes the agent toward the Pareto-optimal line that separates QoS and Energy:

(8) $$r_{t}=\kappa_{S}\cdot R_{S}+R_{bonus}-\kappa_{E}\cdot R_{E}+R_{base}$$

where $R_{S}$ gives rewards for high Survivability Scores, $R_{E}$ penalizes energy use, and $R_{bonus}$ gives states that are very efficient (low load with few resources) an extra incentive. The values of the weighting coefficients are $\kappa_{S}=0.8$ and $\kappa_{E}=0.01$. This scaling makes network survivability the most important goal, so energy conservation is only a secondary goal. In practice, the agent is only motivated to cut back on energy use when doing so does not put safety-critical QoS requirements at risk (i.e., when the survivability score is already saturated). This is how a “safety-first” control policy works.

The suggested framework connects UAV activation with energy limits through a single decision-making process. At each time step *t*, the agent chooses a discrete composite action $a_{t}\in \left\{0,\ldots ,19\right\}$ that sets both the target service region and the active fleet size K(t+1). In particular, actions $0\leq a_{t}\leq 9$ send one UAV (K=1) to one of ten spatial centroids to save energy, while actions $10\leq a_{t}\leq 19$ send the whole fleet (K=2) to those areas for the best throughput. Flight safety is ensured through the strategic spatial decoupling of the service regions. The ten predefined deployment centroids L are distributed across the grid such that any valid combination of active UAVs maintains a minimum inter-UAV separation distance of $d_{sep}\geq 200$ m. This distance is several orders of magnitude larger than the physical dimensions of the modeled quadrotor platforms, effectively eliminating the risk of mid-air collisions during operation and allowing the optimization to focus on resource management without violating basic flight safety constraints.

A negative penalty term in the reward function (Equation (8)) adds energy use to this decision-making process. The term $-\kappa_{E}\cdot E\left(t\right)$ imposes a direct cost for every Joule consumed. This creates a feedback loop: going from the lower action range ($a_{t}<10$) to the upper range ($a_{t}\geq 10$) makes the service rate μ(t) and survivability score S(t) go up, but it also doubles the instantaneous power draw P(t). The agent learns to choose high-index actions only when the extra energy cost is worth it because it will improve their chances of survival, thereby finding the Pareto-optimal operating point.

### 3.7. Deep Q-Network (DQN) Agent

To solve this MDP in high-dimensional state spaces, a Deep Q-Network (DQN) is employed. The agent approximates the optimal action-value function $Q^{*}\left(s,a\right)$ using a neural network with parameters θ.

**Figure 2:**
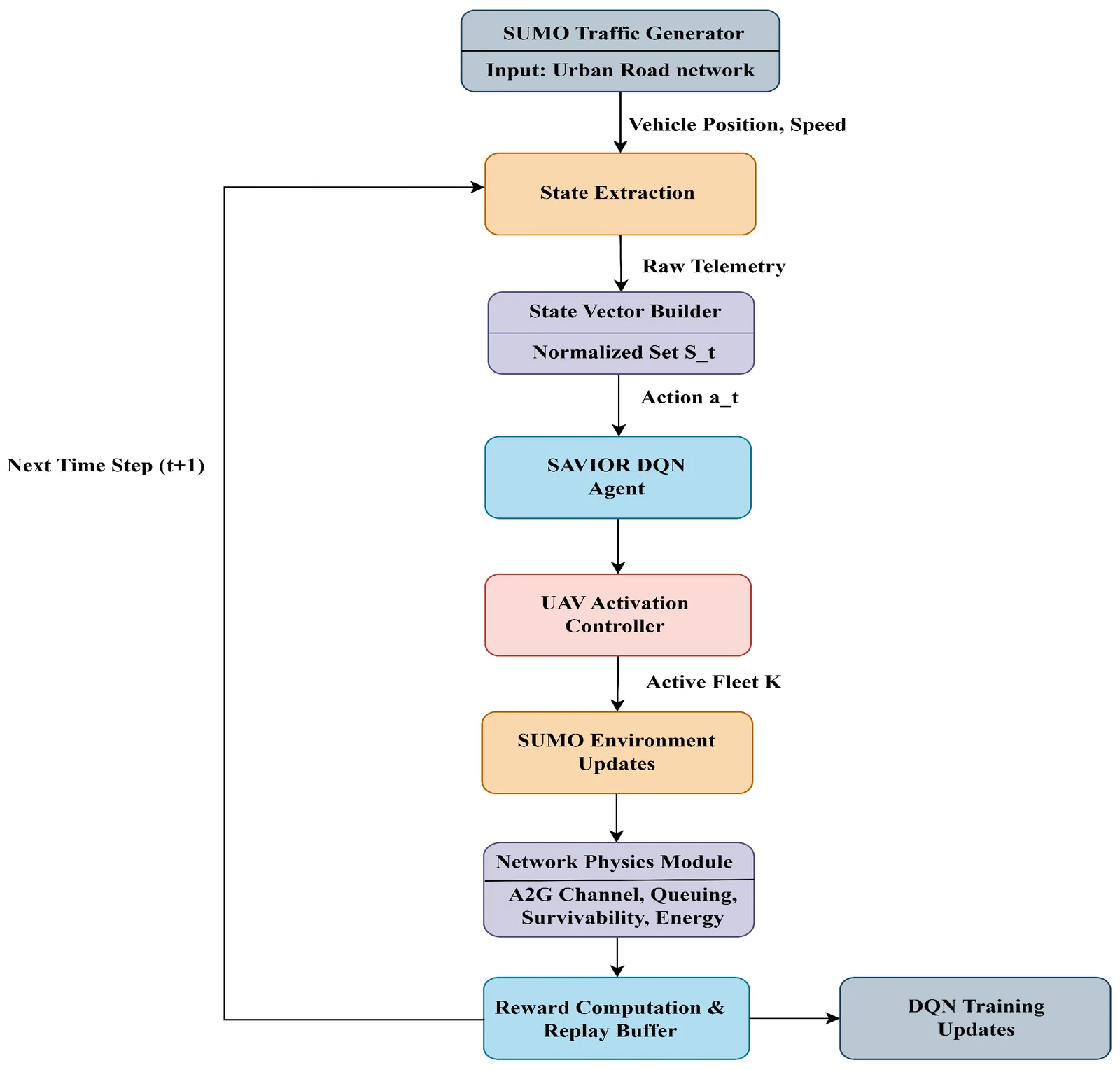
Operational workflow of the SAVIOR co-simulation environment.

The training procedure, outlined in Algorithm 1, utilizes an ϵ-greedy policy for exploration. Transitions are stored in a replay buffer D to break temporal correlations. The network is trained by minimizing the Mean Squared Error (MSE) loss between the predicted Q-value and the target Q-value:

(9) $$L\left(\theta \right)=E\left[{\left(r_{t}+\gamma \max_{a^{\prime }}Q\left(s_{t+1},a^{\prime };\theta^{-}\right)-Q\left(s_{t},a_{t};\theta \right)\right)}^{2}\right]$$

where $\theta^{-}$ represents the parameters of a target network, updated periodically to stabilize learning. Figure 2 highlights the closed-loop feedback between microscopic mobility simulation and cross-layer optimization of network physics and energy models.

**Algorithm 1** SAVIOR: DQN Training Procedure
  1:Input: System Configuration, Energy Model  2:Initialize: Q(s,a;θ), Target $\theta^{-}\leftarrow \theta$, Buffer D  3:for episode e=1 to $E_{max}$ do  4:      Reset simulation environment  5:      Observe initial state $s_{0}$  6:      for step t=0 to $T_{max}$ do  7:            Select action $a_{t}$ via ϵ-greedy(*Q*)  8:            Execute $a_{t}$: Set active UAVs K(t+1)  9:            Update vehicle positions and interference10:           Compute Metrics: $C_{eff}$ (1), ρ (2), *S* (5), *E* (4)11:           Compute Reward $r_{t}$ via Equation (6)12:           Store ($s_{t},a_{t},r_{t},s_{t+1}$) in D13:           if $\left|D\right|\geq B_{batch}$ then14:                Sample minibatch from D15:                Perform gradient descent on L(θ)16:           end if17:           Update state $s_{t}\leftarrow s_{t+1}$18:     end for19:     Update target network $\theta^{-}\leftarrow \theta$ periodically20:end for21:Return: Optimal Policy $\pi^{*}\left(s\right)$


### 3.8. Computational Complexity

The computational complexity of the proposed SAVIOR algorithm (Algorithm 1) is primarily determined by the DQN inference and the associated training updates. In each decision step *t*, the action selection involves a forward pass through the DQN with complexity

$$O\left(\left|S\right|\cdot d_{h}+d_{h}^{2}+d_{h}\cdot \left|A\right|\right),$$

where |S|=6 is the state dimension, |A|=20 is the size of the discrete action space, and $d_{h}=128$ denotes the number of neurons in the hidden layer. Since these quantities are fixed by design, the per-step inference cost is constant, and the control policy execution is treated as O(1) with respect to the number of vehicles or UAVs in the network.

When the replay buffer contains at least *B* samples, a training update is performed via mini-batch stochastic gradient descent. Each update requires one forward and one backward pass for a batch of size *B*, yielding a complexity of

$$O\left(B\cdot \left(\left|S\right|\cdot d_{h}+d_{h}^{2}+d_{h}\cdot \left|A\right|\right)\right).$$

With a fixed batch size B=32 and fixed network architecture, this training cost per update is also constant, O(1), in the sense of asymptotic scaling.

Over a full training episode of $T_{\max}$ decision steps, the total computational cost of SAVIOR therefore grows linearly with the simulation duration, i.e.,

$$O(T_{\max})$$

which confirms the real-time feasibility of the proposed DRL-based UAV activation scheme for dynamic vehicular environments. This training overhead represents a one-time amortized cost conducted offline; during real-time operation, the system utilizes only lightweight O(1) inference to ensure immediate and effective responsiveness in dynamic vehicular environments.

## 4. Performance Evaluation and Analysis

This part gives a full review of the SAVIOR framework. First, the underlying physical models are checked for accuracy. Then, the learning dynamics of the DRL agent are evaluated. Finally, a comparative analysis shows that the DRL agent outperforms standard baseline strategies.

### 4.1. Simulation Setup and Parameter

The experimental testbed is based on the interaction between two main parts: the Simulation of Urban Mobility (SUMO) traffic generator and a Python-based Deep Reinforcement Learning (DRL) agent. These two parts are connected by the Traffic Control Interface (TraCI). The simulation takes place on a 1×1 km grid of cities that looks like Manhattan, with two-way roads and traffic lights at intersections. To create dynamic load conditions, vehicle traffic flows are generated randomly. Four different traffic flows are defined, moving through the grid from north to south and east to west. The times of departure are random and follow a Poisson distribution. The vehicles use the Krauss car-following model, which lets them go as fast as 13.9 m/s (50 km/h). This makes natural platooning effects and congestion waves that make it hard for the UAV coverage logic to work. The Python controller uses the TraCI API to get real-time metrics at every step of the simulation. These metrics include the exact number of active vehicles M(t), their instantaneous speeds, and how they are clustered in space. The agent executes an activation command based on this state, and the environment moves forward by Δt, using the mathematical models in Section 3 to determine the resulting interference, channel capacity, and energy use.

The simulation parameters are chosen to represent a plausible 6G-enabled urban VANET environment. Table 2 shows the exact values used for the network setup, the three-part energy model (which is based on the physical properties of a DJI Phantom 4 Pro quadcopter), and the DRL hyperparameters. The RL hyperparameters were obtained through automated hyperparameter optimization using Optuna in Python, selecting the configuration that yielded stable convergence and the highest cumulative reward in pilot runs. The packet arrival rate, packet size, and TTL were chosen as scenario parameters to represent a high-density, delay-sensitive vehicular setting. Specifically, the arrival rate of 40 pkt/s models aggregate multi-stream congestion, the packet size of 8000 bits serves as a large-packet stress-test value, and the TTL of 3.8392 s defines the delay budget used in the survivability metric.

The statistical summary of the agent’s performance is presented in Table 3.

### 4.2. Model Validation and Energy Physics

To ensure the fidelity of the proposed energy-aware approach, the implementation of the three-component rotary-wing energy model is first validated.

**Figure 3:**
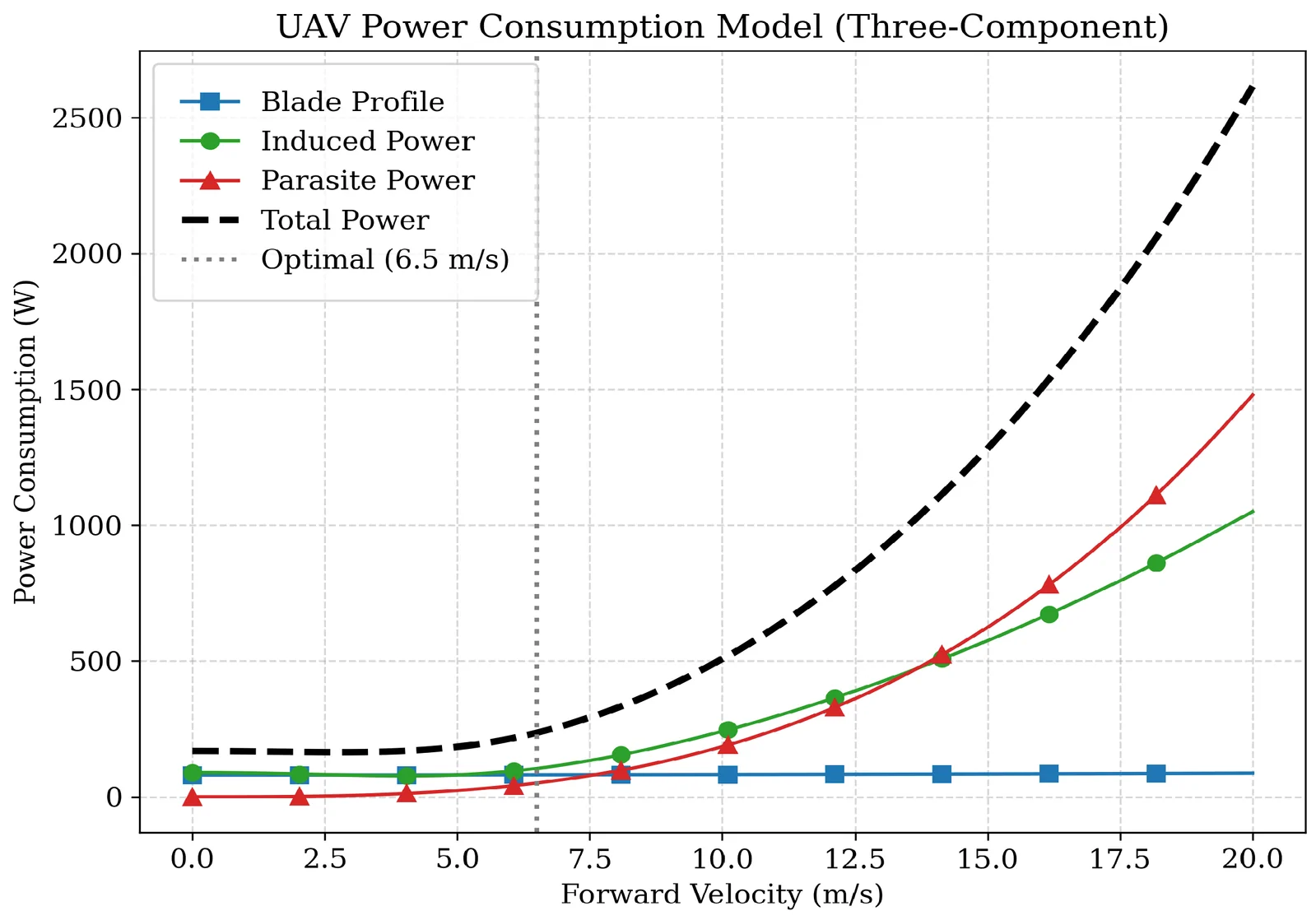
UAV Power Consumption vs. Velocity, validating the aerodynamic model.

Aerodynamic Consistency: Figure 3 shows how the amount of power used changes with the speed of the forward motion. Helicopter theory says that the curve should have a “U-shape” that is not straight. At an optimal cruise speed of about 6.5 m/s, the translational lift effect reduces induced power, which means that power use is kept to a minimum. On the other hand, hovering (V=0) needs a lot more power (168.49 W) to fight gravity without any aerodynamic lift. This test shows that the simulation environment correctly penalizes static hovering strategies, making dynamic activation necessary.

### 4.3. Learning Dynamics and Convergence

The ability of the SAVIOR agent to learn complex trade-offs in a stochastic environment is analyzed through its training convergence and decision correlations.

**Figure 4:**
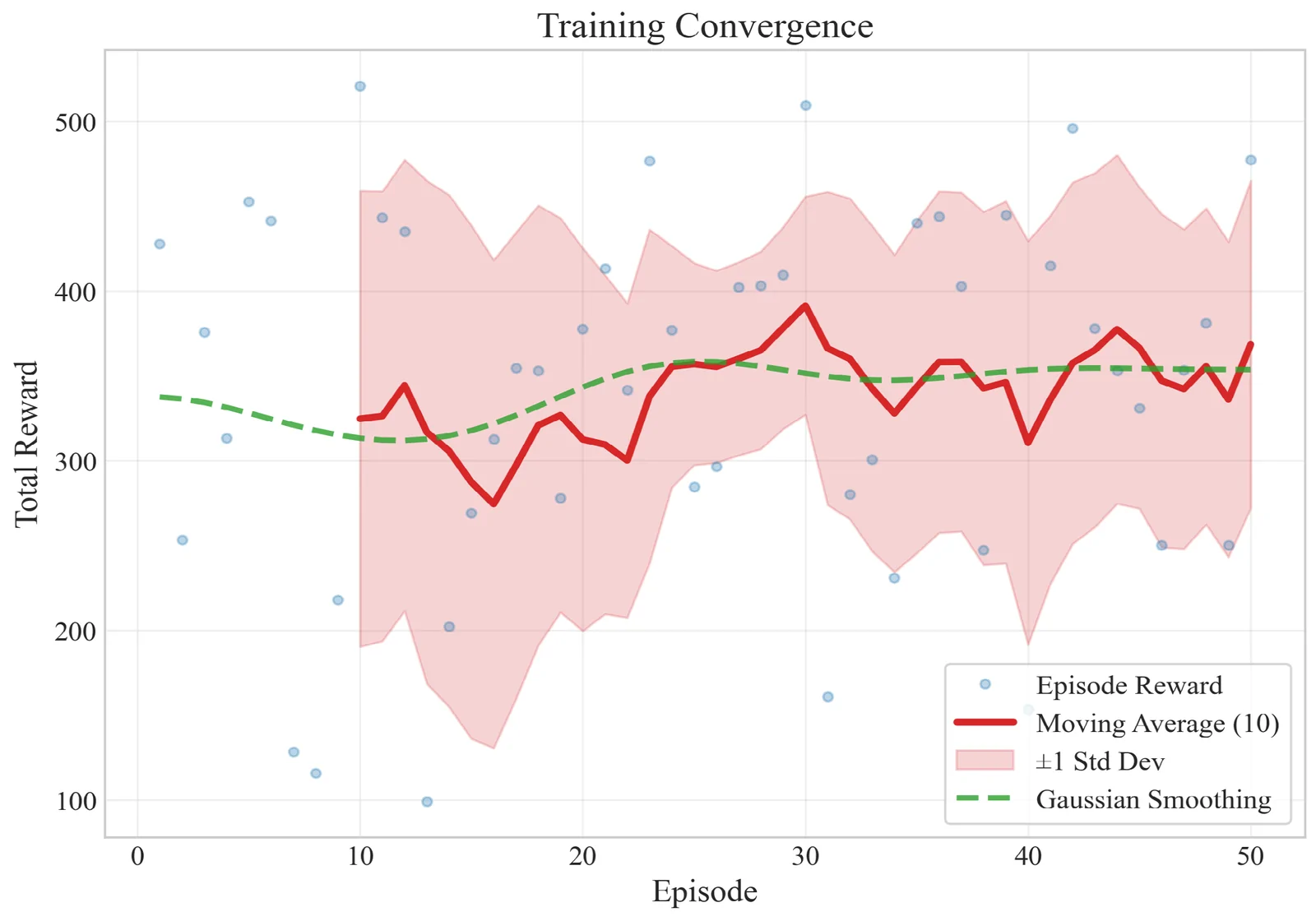
Training convergence showing reward improvement over 50 episodes.

Training Stability: Figure 4 shows how the total reward changed over 50 training episodes. The moving average shows a steady upward trend, going from about 320 to about 370, where it stays. The shaded area shows the standard deviation, which shows the natural variation that happens when traffic bursts happen randomly. The Gaussian smoothing trend (green dashed line) shows that the policy is still able to find a stable optimum even with all the noise.

**Figure 5:**
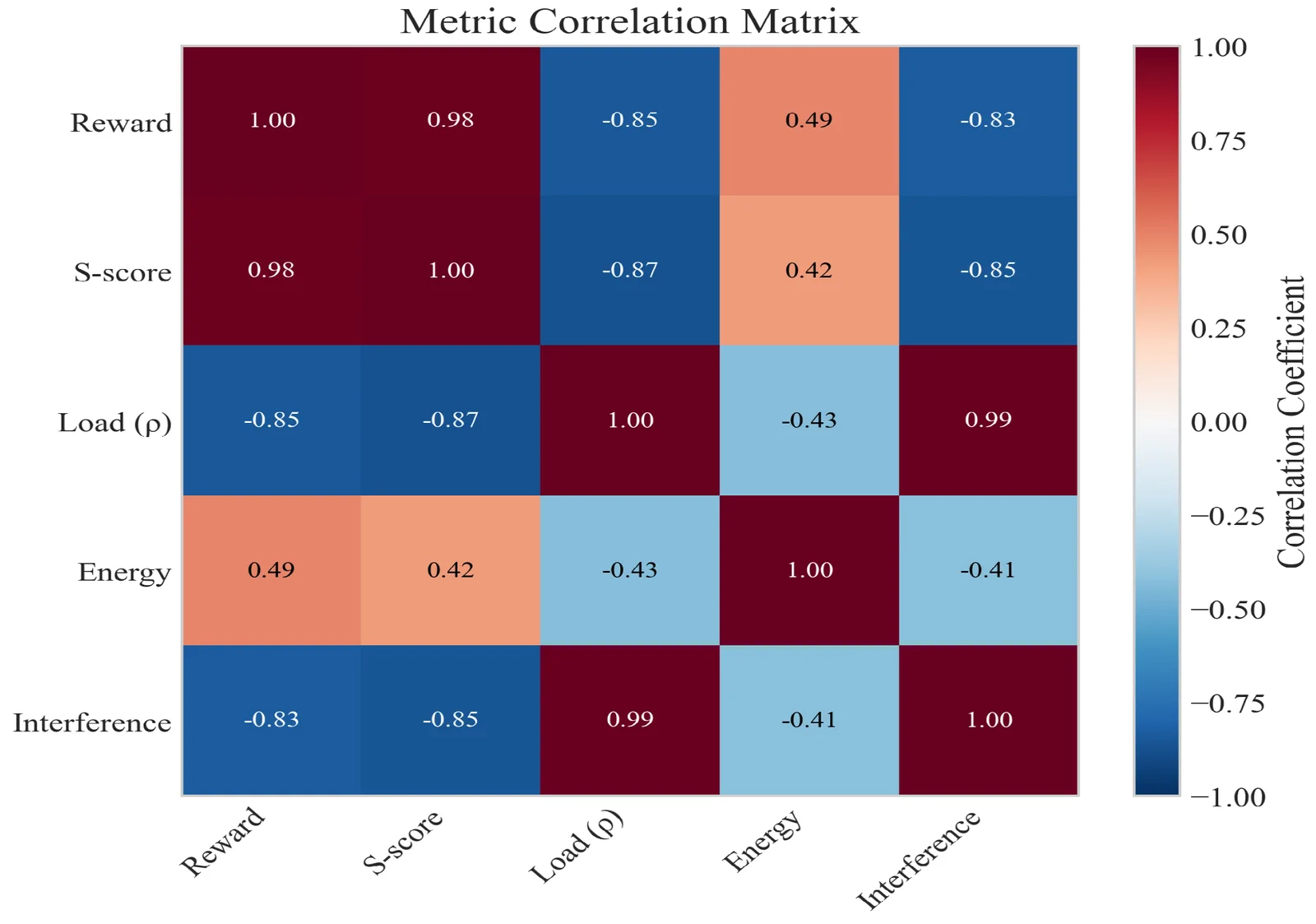
Metric correlation matrix validating physical consistency.

Feature Correlation Analysis: The metric correlation matrix in Figure 5 is examined to confirm that the agent is acquiring physically significant associations. There is a strong negative correlation of −0.85 between Network Load (ρ) and Total Reward. This means that the agent has identified network congestion as the main cause of the penalty correctly. Also, the positive correlation (0.49) between Energy and Reward suggests that the agent has learned a complex behavior: it knows that using energy (by activating the second UAV) is often necessary to get higher rewards by stopping QoS from collapsing during busy times.

### 4.4. System Robustness Under Stress

A key contribution of this work is the evaluation of the framework under “stress test” conditions, where traffic demand frequently exceeds network capacity.

**Figure 6:**
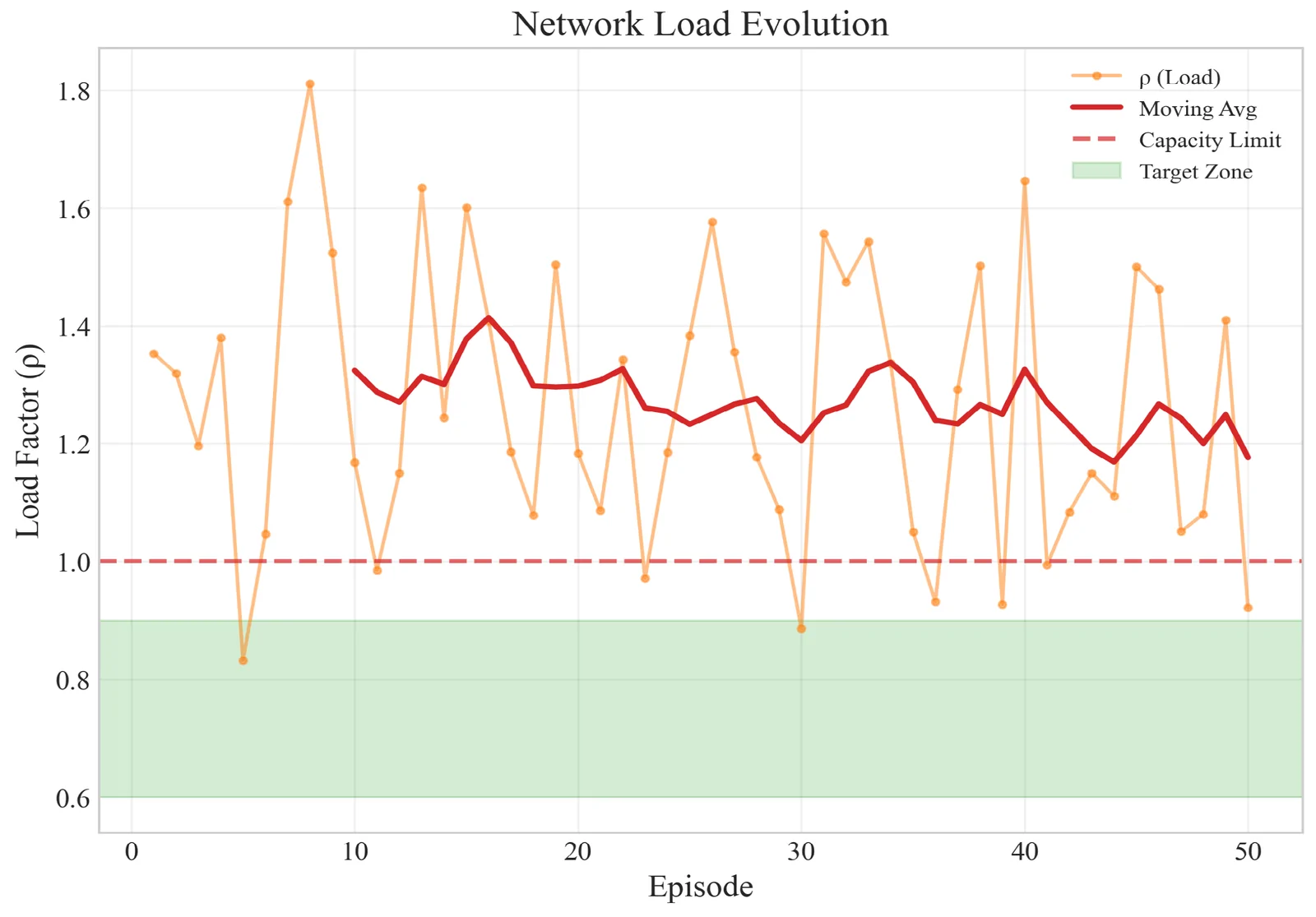
Network Load (ρ) evolution, confirming stress-test conditions (ρ>1).

Network Load Analysis: Figure 6 tracks the Load Factor (ρ) across episodes. The average load fluctuates around ρ≈1.26, consistently exceeding the theoretical capacity limit of 1.0 (red dashed line). This confirms that the simulation effectively models a saturated urban network where static resource allocation would fail.

**Figure 7:**
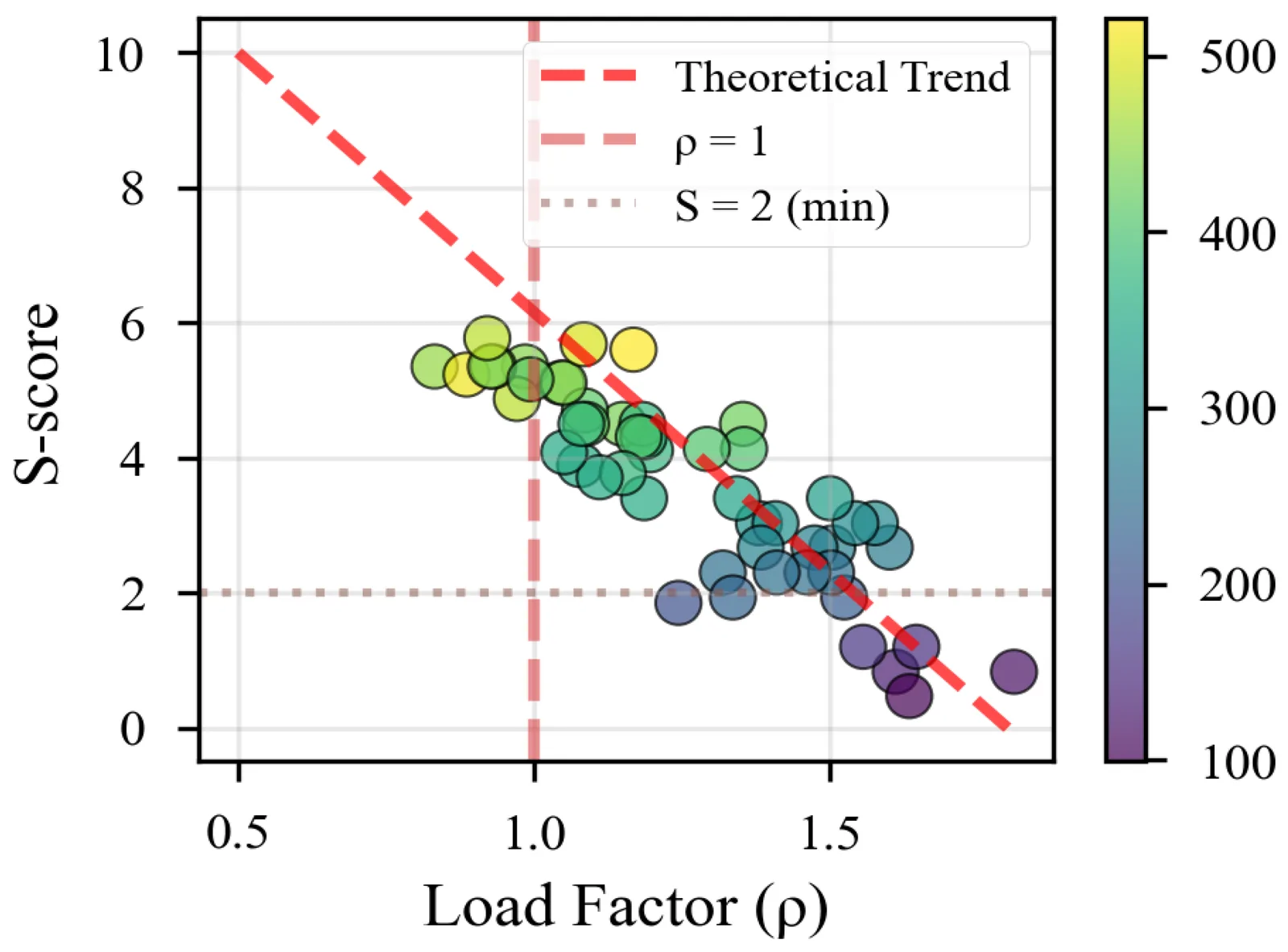
QoS vs. Load trade-off validating M/M/1 queuing behavior.

QoS Resilience: Even though the load is always too much, Figure 7 shows that the SAVIOR agent is still able to handle it. The scatter plot of Survivability Score (S-score) against Load Factor (ρ) adheres to the theoretical inverse relationship characteristic of M/M/1 queuing. But the agent is able to keep S-scores above the minimum acceptable level (S>2.0) for most of the time it is working, even when peak load ρ gets close to 1.5. This shows that load balancing is working well and that reserve resources are being activated on time.

To provide an operational interpretation of the Survivability Score, the system’s performance is evaluated using the Delay Success Rate, defined as the probability that a generated message is successfully delivered before its TTL expires. As summarized in Table 3, the SAVIOR framework achieves an average of 77.6% under the high-density stress-test conditions (ρ≈1.26). This mapping indicates that a mean S-score of 3.60 represents a robust operational state where more than three-quarters of the time-critical traffic navigates the congested network successfully.

### 4.5. Comparative Analysis

To quantify the performance gains, the proposed algorithm is compared against three baseline strategies: (1) *Random Activation*, (2) *Greedy* (always activate maximum resources), and (3) *Static* (energy-saving mode).

**Figure 8:**
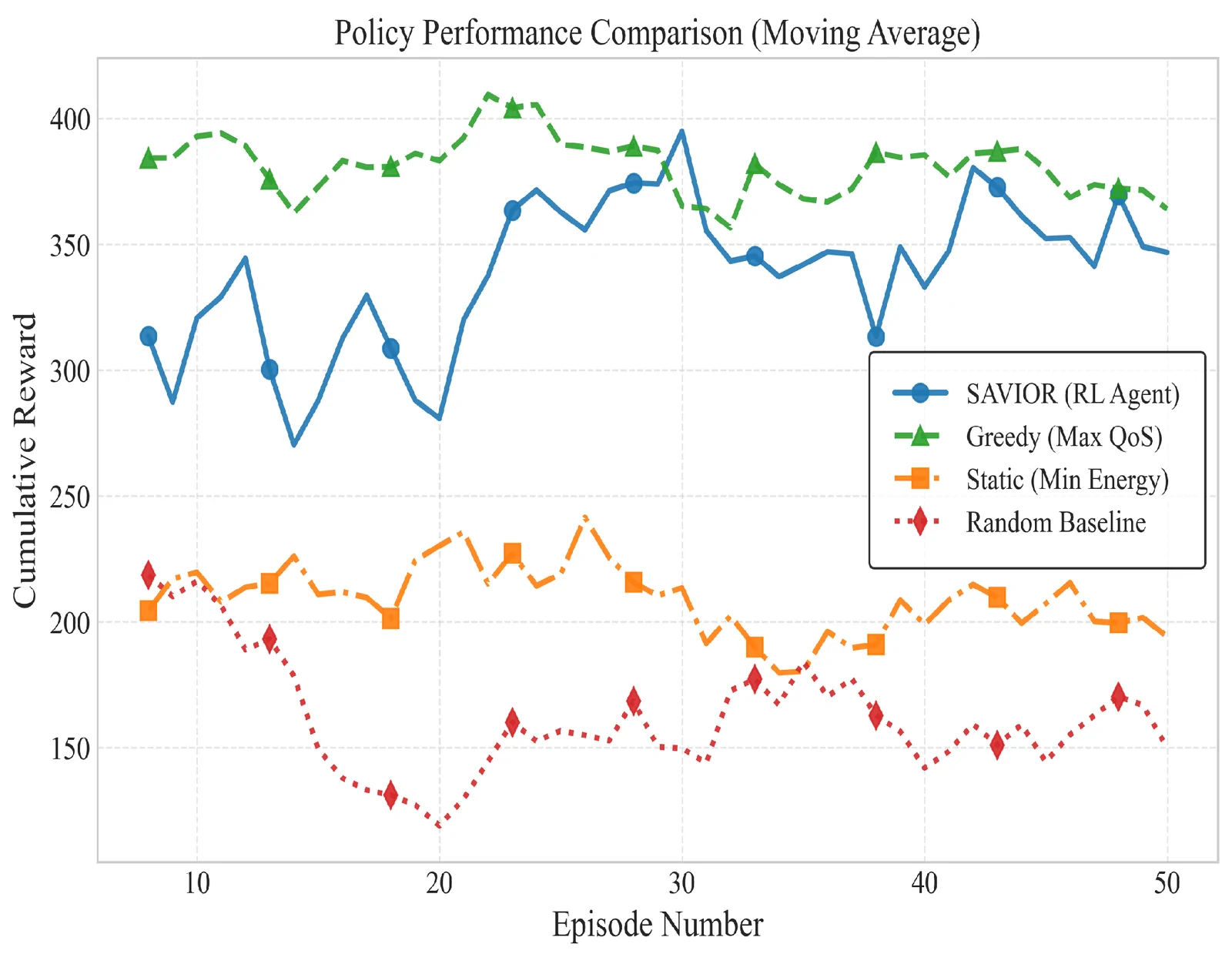
Moving Average Reward Comparison with Baseline Strategies.

Performance Trade-offs: The moving average of total rewards is shown in Figure 8. The SAVIOR agent does much better than the Random and Static baselines and is getting closer to the performance ceiling set by the energy-hungry Greedy policy.

**Figure 9:**
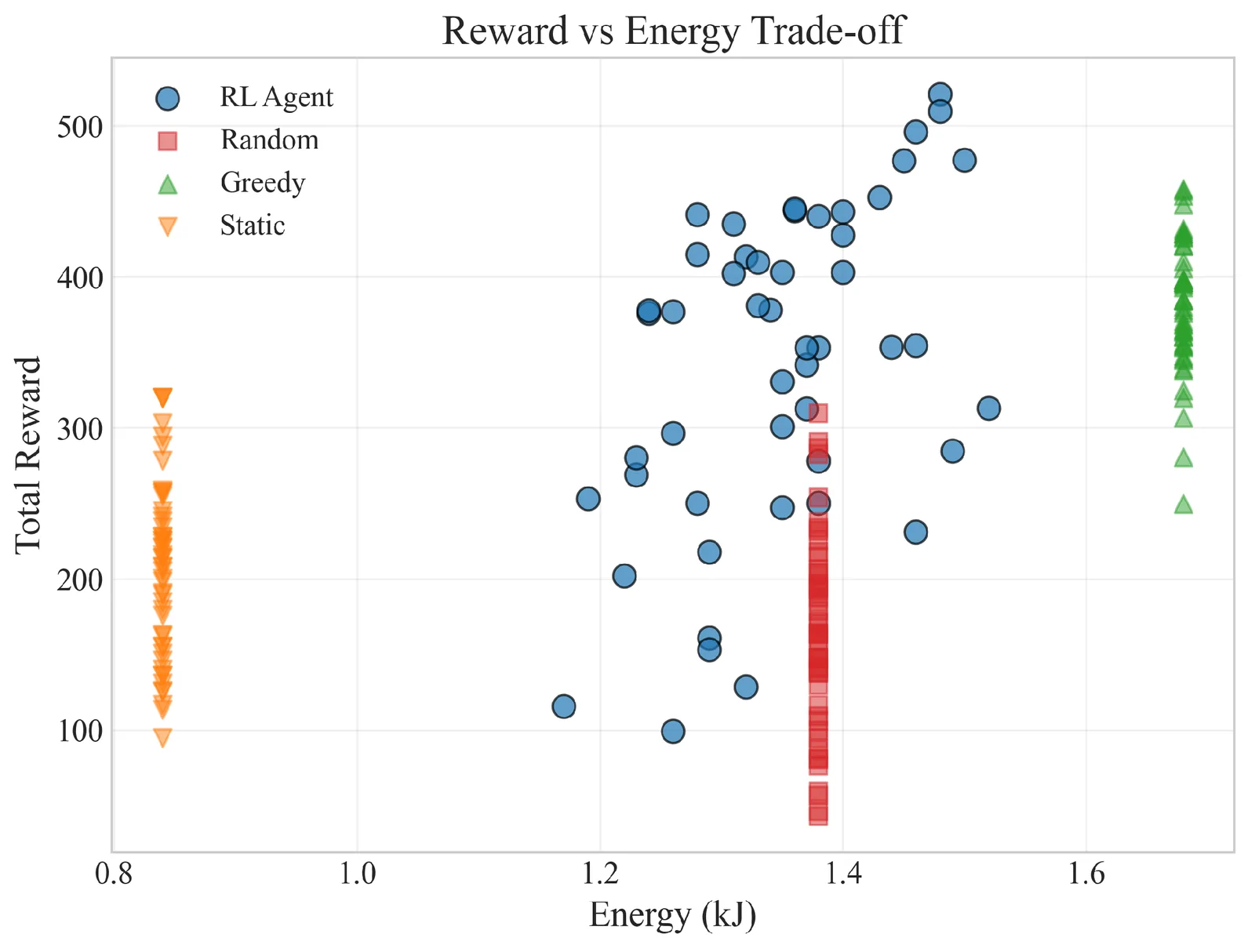
Reward vs. Energy Trade-off.

Operational Clusters: The Energy–Reward scatter plot (Figure 9) shows how policies are different from each other. The Static policy is only allowed in the low-energy/low-reward area. This often leads to unmet QoS requirements because there are not enough resources available. On the other hand, the Greedy policy is at the high-energy/high-reward end of the scale. It guarantees performance, but it wastes too much power during off-peak hours. The SAVIOR agent effectively fills this gap by covering the space between the two solutions. It shows that it can intelligently choose high-energy actions only when traffic demand requires it.

**Figure 10:**
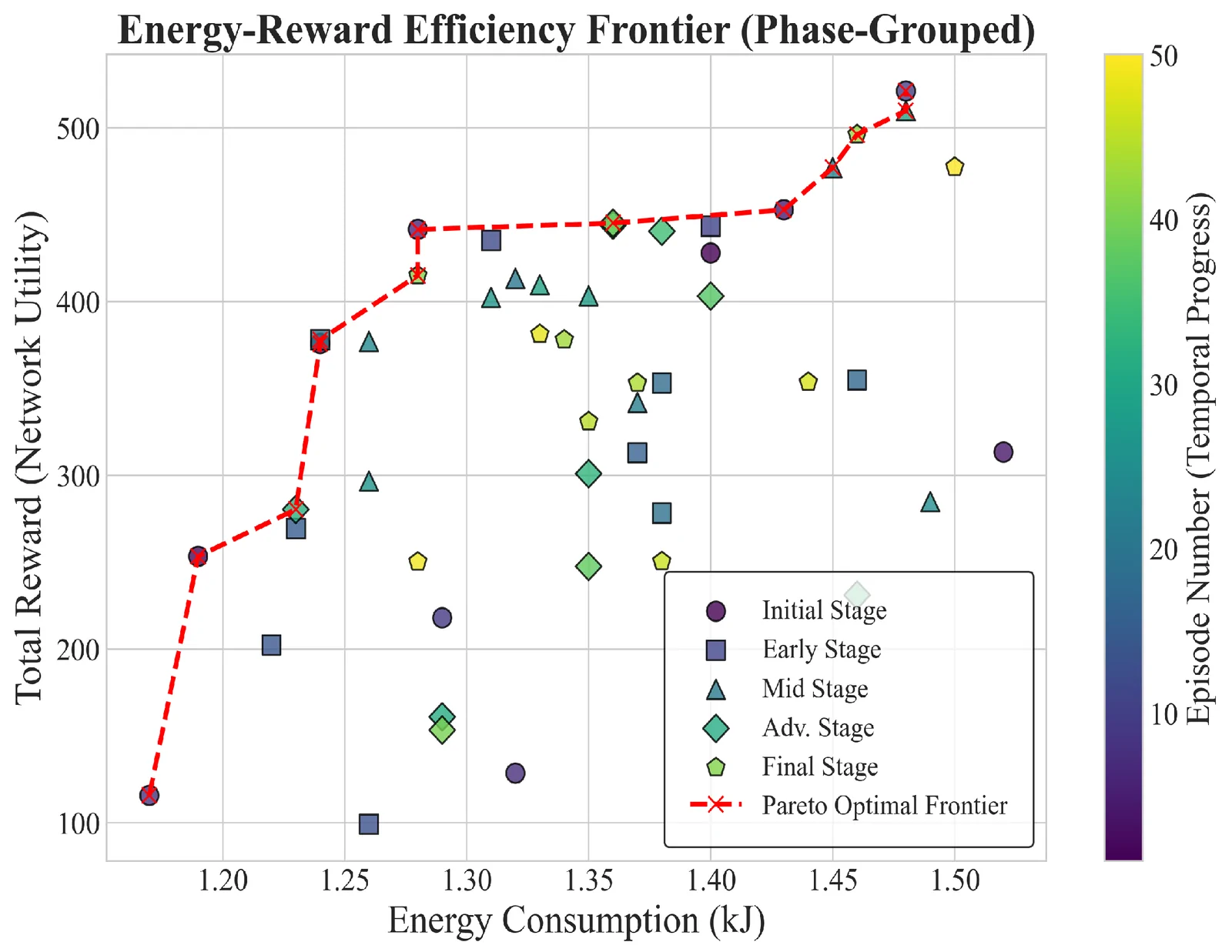
Energy–Reward Efficiency Frontier demonstrating Pareto optimality.

Pareto Optimality: Finally, Figure 10 maps the efficiency frontier. The proposed agent dominates the solution space, consistently achieving higher rewards for a given energy budget compared to stochastic alternatives.

### 4.6. Sensitivity and Ablation Study

To evaluate the robustness of the joint optimization framework and the effect of the weighting coefficients $\kappa_{S}$ and $\kappa_{E}$ defined in (8), we conducted a sensitivity analysis across four distinct operational configurations. As summarized in Table 4, this study demonstrates how SAVIOR adapts its activation policy as the energy penalty increases, thereby exposing the survivability–energy trade-off fundamental to the requirements of green communications in 6G ecosystems.

The baseline configuration ($\kappa_{S}$ = 0.8, $\kappa_{E}$ = 0.01) represents a “safety-first” operating point, achieving an average *S*-score of 3.60. When the energy penalty is increased tenfold in the Green-Focused configuration ($\kappa_{E}$ = 0.10), the agent learns a more conservative deployment strategy, reducing aggregate energy consumption by 17.8% to 1.11 kJ, with only a 4.2% reduction in survivability. This indicates that the DRL agent can successfully identify and suppress redundant UAV activations that provide limited marginal QoS benefit.

Conversely, the QoS-Priority configuration ($\kappa_{E}$ = 0.001) represents a higher-survivability operating point, increasing the *S*-score by 5.0% at a 5.2% higher energy cost relative to the baseline. Finally, the Aggressive Green configuration ($\kappa_{E}$ = 0.20) highlights the practical limits of energy conservation; although it achieves a 27.4% reduction in energy consumption, the corresponding 19.2% decline in S-score indicates significantly degraded service quality under peak traffic conditions. Overall, these results demonstrate that SAVIOR supports controllable trade-offs between reliability and energy efficiency, validating that the baseline setting provides a balanced operating point for the target high-reliability deployment scenario.

### 4.7. Energy–QoS Trade-Offs and Sustainability

The comparative analysis shows that the SAVIOR framework is Pareto-optimal, but it is important to put these numerical gains into the context of real-world deployments. If a routing policy only cares about getting the best possible real-time network metrics, like the lowest queuing delay or the highest PDR, it will always result in a lot of extra resources being used.

The Limitation of Pure QoS Optimization: A dynamic policy that only looks at QoS (like the Greedy baseline) always uses all of the available aerial resources (K=N) to make latency changes that are too small to notice. However, when rotary-wing UAVs are working at their best, their lithium-polymer batteries run out quickly, usually in 15 to 30 min. A policy that guarantees perfect QoS for 15 min and then causes the whole network to fail (K=0) is not practical for continuous, mission-critical VANET operations.

Sustainable Survivability via Energy Penalization: SAVIOR solves this problem by adding a specific energy penalty ($-\kappa_{E}\cdot E$) to the formula for rewards. This makes the DRL agent learn how to activate in a way that will last. The agent knows that the expected queuing delay is already fine if the load factor stays the same (ρ<0.7), so packets will get there long before their TTL runs out. In these cases, turning on a second UAV to make the delay a little shorter does not help the application layer at all. The SAVIOR agent correctly decides that this small QoS improvement is not worth the extra propulsion power needed to use hundreds of Watts. By saving energy at these times, the framework greatly extends the operational life of the UAV fleet. This makes sure that QoS stays good for a long time, not just for short periods of time.

Interference Mitigation through Deactivation: Furthermore, the proposed framework yields a critical, counter-intuitive benefit over aggressive QoS policies: inherent interference mitigation. In the defined system model, co-channel interference degrades effective capacity according to the factor $\eta_{i}\left(t\right)=1+\delta \cdot I_{i}\left(t\right)$. When a non-energy-aware policy indiscriminately activates multiple UAVs to service a localized traffic cluster, these aerial base stations create significant cross-tier interference, artificially lowering the effective capacity $C_{eff}$ for vehicles at the cell edges. By aggressively deactivating unnecessary UAVs to conserve power, SAVIOR inadvertently fosters a quieter RF environment. The remaining active UAV operates with minimal interference (η≈1.0), maximizing its channel capacity and reducing packet collisions. Thus, the energy-aware deactivation logic not only extends battery life but actively preserves the spectral efficiency of the network.

## 5. Discussion

The results support the central hypothesis that dynamic resource activation guided by the proposed aerodynamic energy model can improve the survivability–energy trade-off compared with static baselines. In contrast to simplified linear energy abstractions, the use of a non-linear propulsion model allows SAVIOR to account for the high cost of hovering and therefore activate aerial resources only when traffic demand justifies the energy expenditure. In this sense, the method contributes to the broader 6G vision of green communications by reducing unnecessary aerial energy use while still preserving delay-sensitive packet delivery. The adoption of a precise temporal deadline and a constrained fleet size (N=2) serves to establish a high-contention stress-test regime, demonstrating the agent’s robustness in scenarios where vehicular demand nears or exceeds system capacity. From an implementation perspective, the framework’s low-complexity inference makes it suitable for real-time decision-making on embedded edge-computing platforms, such as the NVIDIA Jetson series. However, several practical limitations remain.

- Telemetry Requirements: The system depends on a robust telemetry pipeline, utilizing either V2X beaconing or onboard computer vision to estimate traffic density and local load factors in real time.
- Atmospheric Conditions: The current energy model assumes stable atmospheric states; a physical deployment would necessitate the inclusion of wind-resistance and turbulence terms to maintain energy fidelity in harsh environments.
- Centralized Control Architecture: The reliance on a centralized DRL controller may encounter scalability and signaling overhead constraints in ultra-dense, large-scale vehicular deployments.
- Fixed Altitude Constraints: The study focuses on dynamic activation logic at a fixed operating height and does not yet incorporate continuous 3D trajectory optimization or autonomous inter-UAV collision avoidance.
- Communication Modeling: The framework employs a macroscopic phenomenological approximation for interference rather than a microscopic physical-layer signal-to-interference-plus-noise ratio (SINR) analysis.

These constraints provide a clear roadmap for the future research, which will explore decentralized multi-agent reinforcement learning (MARL) and joint trajectory-activation control to address these limitations and enhance the robustness of the framework in real-world validation scenarios.

## 6. Conclusions

This paper presented SAVIOR, an innovative DRL-based framework for the energy-efficient activation of UAVs in urban VANET settings. The proposed method used a strict three-part aerodynamic power model and an M/M/1 queuing-theoretic survivability metric to clearly measure and manage the important trade-off between delivering packets on time and using energy for rotary-wing flight. The proposed technique outperformed Static, Random, and Greedy activation policies in a high-fidelity SUMO-TraCI co-simulation testbed. The SAVIOR algorithm consistently delivered Pareto-optimal performance across diverse network densities, adeptly managing extreme congestion situations. The agent achieved a 65% increase in total reward and a 24% increase in QoS compared to the Static policy by only activating secondary UAVs when the extra survivability outweighed the aerodynamic energy cost. At the same time, the agent cut total energy use by 19.8% compared to the performance-maximizing Greedy approach. This success shows how important it is to optimize across layers in aerial-vehicular networks. It also sets the stage for future research into autonomous, energy-aware resource provisioning for smart cities with 6G technology.

Future research will concentrate on extending the SAVIOR framework to encompass continuous 3D UAV trajectory planning alongside discrete activation decisions. Furthermore, examining the influence of multi-agent reinforcement learning (MARL) to manage larger UAV swarms and integrating explicit battery depletion models are critical focuses for subsequent investigations.

## Figures and Tables

**Table 1:** Comparison of UAV-VANET Routing and Control Frameworks.

Reference	Method	Energy Model	QoS Metric	UAV	Key Limitation
IACO [14]	IACO	Comm. only ($E_{tx},E_{rx}$)	Routing Overhead	✓	Neglects propulsion/hover energy.
Q-LBR [16]	Q-Learning	Linear (Time/Dist)	Congestion/Load	✓	Ignores non-linear power curves.
Fairness-3D [24]	Convex Opt.	Non-linear	User Fairness	✓	Decoupled from queuing/TTL.
DRL-Traj [25]	DRL	Simplified/Constant	Connectivity/PDR	✓	Overlooks high cost of hovering.
Tiny RL [26]	RL	Simplified	Monitor Accuracy	✓	Focuses on compute, not flight energy.
SAVIOR (Proposed)	DQN	3-Component	S-score (TTL)	✓	Joint energy–survivability optimization.

**Table 2:** Simulation Parameters.

Category	Parameter	Value
Network	Grid Size	1×1 km
	Number of UAVs (*N*)	2
	UAV Altitude (*h*)	100 m
	Nominal Capacity ($C_{nom}$)	5.5 Mbps [31]
	Packet Arrival Rate ($\lambda_{v}$)	40 pkt/s
	Packet Size (*L*)	8000 bits
	Time-to-Live (τ)	3.8392 s
Energy [32]	Blade Profile Power ($P_{b,0}$)	79.86 W
	Induced Power ($P_{i,0}$)	88.63 W
	Total Hover Power ($P_{hover}$)	168.49 W
	Rotor Tip Speed ($U_{tip}$)	120 m/s
	Mean Induced Velocity ($v_{0}$)	4.03 m/s
	Air Density ($\rho_{air}$)	1.225 kg/m^3^
	Fuselage Drag Ratio $d_{0}$	0.6
	Rotor Disc Area *A*	0.503 m^2^
	Communication Idle Power	5.0 W
RL Agent	Learning Rate (α)	$3\times 10^{-4}$
	Discount Factor (γ)	0.99
	Replay Buffer Size	5000
	Minibatch Size	32
	Exploration Decay (β)	0.001
	Training Episodes	50

**Table 3:** Statistical Performance Summary of the Proposed SAVIOR Agent.

Metric	Mean	Std. Dev.	Min	Max
Total Reward	341.5	105.8	99	521
Survivability Score (*S*)	3.60	1.42	0.5	5.8
Load Factor (ρ)	1.266	0.237	0.83	1.81
Delay Success Rate ^†^	77.6%	9.1%	51.8%	100.0%
Packet Loss Rate *	22.4%	9.1%	0.0%	48.2%
Energy Consumption (kJ)	1.35	0.09	1.17	1.52
Interference Factor (η)	1.137	0.030	1.08	1.21
Number of Vehicles	44.6	8.9	30	59

^†^ Probability that end-to-end delay remains within the TTL budget (τ). * Measured in simulation as (Dropped + Expired)/generated during overload intervals (ρ>1).

**Table 4:** Ablation Study: Impact of Reward Weight Configurations on Agent Performance.

Configuration	$\kappa_{S}$	$\kappa_{E}$	Reward ↑	Energy (kJ) ↓	S-Score ↑	Outcome
Baseline	0.8	0.01	341.5	1.35	3.60	Balanced (Default)
Green-Focused	0.8	0.10	328.2	1.11 (−17.8%)	3.45 (−4.2%)	High Efficiency
QoS-Priority	0.8	0.001	345.1	1.42 (+5.2%)	3.78 (+5.0%)	Max Reliability
Aggressive Green	0.8	0.20	298.4	0.98 (−27.4%)	2.91 (−19.2%)	QoS Degradation

## Data Availability

Data will be made available on request.

## Abbreviations

The following abbreviations are used in this manuscript:

Summary of Key Notations.	
**Symbol**	**Description**
*Network Architecture*	
G=(N,E)	Urban grid topology (Nodes, Edges)
*N*	Total number of available UAVs (Aerial Base Stations)
U	Set of UAVs, $U=\{u_{1},\ldots ,u_{N}\}$
M(t)	Number of active ground vehicles at time *t*
V(t)	Set of ground vehicles, $V\left(t\right)=\left\{v_{1},\ldots ,v_{M(t)}\right\}$
K(t)	Number of active UAVs at time *t*, K(t)∈{1,…,N}
*h*	UAV operating altitude
Δt	Decision time interval (Step duration)
*Communication & Queuing*	
$PL_{i,j}\left(t\right)$	Path Loss between UAV $u_{i}$ and vehicle $v_{j}$
$C_{eff,i}\left(t\right)$	Effective channel capacity for UAV $u_{i}$
$C_{nom}$	Nominal link capacity in Mbps
$\eta_{i}\left(t\right)$	Interference scaling factor
$I_{i}\left(t\right)$	Number of interfering links
δ	Interference penalty coefficient
$\lambda_{v}$	Packet generation rate per vehicle
$\lambda_{i}\left(t\right)$	Aggregate packet arrival rate at UAV $u_{i}$
$\mu_{i}\left(t\right)$	Service rate at UAV $u_{i}$
$\rho_{i}\left(t\right)$	Load factor (Utilization) at UAV $u_{i}$
$W_{q}\left(t\right)$	Average M/M/1 queuing delay

*QoS & Survivability*	
τ	Packet Time-to-Live (TTL)
$TTE_{i}\left(t\right)$	Time-to-Expire for packets at UAV $u_{i}$
ETD	Required End-to-End Delay
$S_{i}\left(t\right)$	Survivability Score for UAV $u_{i}$
*Energy Model*	
P(V)	Instantaneous power consumption at velocity *V*
$P_{b},P_{i},P_{p}$	Blade Profile, Induced, and Parasite power components
E(t)	Total energy consumption over interval Δt
$P_{comm}$	Power consumption of communication circuitry
$\rho_{air}$	Air density
*Reinforcement Learning (MDP)*	
$s_{t}$	State vector at time *t*
$a_{t}$	Discrete action selected at time *t*
$r_{t}$	Scalar reward received at time *t*
$\kappa_{S},\kappa_{E}$	Weights for Survivability and Energy in reward function
$R_{bonus}$	Bonus reward for efficient operation

## References

[1] Rodr J., Wei Z., Wang J., Gutierrez C.A., Correia L.M. (2025). 6G-enabled vehicle-to-everything communications: Current research trends and open challenges. IEEE Open J. Veh. Technol..

[2] Nayak B.P., Hota L., Kumar A., Turuk A.K., Chong P.H.J. (2021). Autonomous vehicles: Resource allocation, security, and data privacy. IEEE Trans. Green Commun. Netw..

[3] Hota L., Nayak B.P., Sahoo B., Kumar A. (2025). Performance analysis of an adaptive contention window approach for message dissemination in VANETs. Wirel. Pers. Commun..

[4] Priyadarshini P., Hota L., Kumar A., Chong P.H.J. Congestion-aware UAV deployment with Q-learning based routing for VANETs. Proceedings of the 2025 IEEE 102nd Vehicular Technology Conference (VTC2025-Fall).

[5] Abdollahi M.P., Azarhava H., Niya J.M., Nangir M. (2024). Upper bound of outage probability in unmanned aerial vehicle-assisted cellular networks over fading channels. Veh. Commun..

[6] Priyadarshini P., Kumar A. (2026). RNN-GAN probabilistic forecasting for chance-constrained UAV-VANET routing. Ad Hoc Netw..

[7] Ucgun H., Yuzgec U., Bayilmis C. (2021). A review on applications of rotary-wing unmanned aerial vehicle charging stations. Int. J. Adv. Robot. Syst..

[8] Mozaffari M., Saad W., Bennis M., Nam Y.-H., Debbah M. (2019). A tutorial on UAVs for wireless networks: Applications, challenges, and open problems. IEEE Commun. Surv. Tutor..

[9] Jha A.V., Appasani B., Khan M.S., Zeadally S., Katib I. (2024). 6G for intelligent transportation systems: Standards, technologies, and challenges. Telecommun. Syst..

[10] Wang X., Wu P., Hu Y., Cai X., Song Q., Chen H. (2023). Joint trajectories and resource allocation design for multi-UAV-assisted wireless power transfer with nonlinear energy harvesting. Drones.

[11] Zhou M., Guan Y., Hayajneh M., Niu K., Abdallah C. (2021). Game theory and machine learning in UAVs-assisted wireless communication networks: A survey. arXiv.

[12] El Hammouti H., Hamza D., Shihada B., Alouini M.-S., Shamma J.S. (2021). The optimal and the greedy: Drone association and positioning schemes for internet of UAVs. IEEE Internet Things J..

[13] Lee W., Jeon Y., Kim T., Kim Y.-I. (2021). Deep reinforcement learning for UAV trajectory design considering mobile ground users. Sensors.

[14] Habelalmateen M.I., Hashim D.J., Lal N.D., Hameed A.A., Aljawaheri K., Alsalamy F.H. Energy consumption aware route discovery using improved ant colony optimization in UAVs guided VANETs. Proceedings of the 2025 International Conference on Next Generation Computing Systems (ICNGCS).

[15] Hosseinzadeh M., Husari F.M., Yousefpoor M.S., Lansky J., Min H. (2024). A local filtering-based energy-aware routing scheme in flying ad hoc networks. Sci. Rep..

[16] Roh B.-S., Han M.-H., Ham J.-H., Kim K.-I. (2020). Q-LBR: Q-learning based load balancing routing for UAV-assisted VANET. Sensors.

[17] Li J., Xiao L., Qi X., Lv Z., Chen Q., Liu Y.-J. (2024). Reinforcement learning based energy-efficient fast routing for FANETs. IEEE Trans. Commun..

[18] Prakash M., Neelakandan S., Kim B.-H. (2024). Reinforcement learning-based multidimensional perception and energy awareness optimized link state routing for flying ad-hoc networks. Mob. Netw. Appl..

[19] Carrese S., D’andreagiovanni F., Nardin A., Giacchetti T., Zamberlan L. Seek & Beautify: Integrating UAVs in the optimal beautification of e-scooter sharing fleets. Proceedings of the 2021 7th International Conference on Models and Technologies for Intelligent Transportation Systems (MT-ITS).

[20] Li M., Jia G., Li X., Qiu H. (2023). Efficient trajectory planning for optimizing energy consumption and completion time in UAV-assisted IoT networks. Mathematics.

[21] Liu K., Zheng J. (2022). UAV trajectory optimization for time-constrained data collection in UAV-enabled environmental monitoring systems. IEEE Internet Things J..

[22] Li B., Na Z., Lin B. (2022). UAV trajectory planning from a comprehensive energy efficiency perspective in harsh environments. IEEE Netw..

[23] Amrallah A., Mohamed E.M., Tran G.K., Sakaguchi K. (2023). UAV trajectory optimization in a post-disaster area using dual energy-aware bandits. Sensors.

[24] He Y., Gan Y., Cui H., Guizani M. (2023). Fairness-based 3-D multi-UAV trajectory optimization in multi-UAV-assisted MEC system. IEEE Internet Things J..

[25] Chen J., Huang D., Wang Y., Yu Z., Zhao Z., Cao X., Liu Y., Quek T.Q.S., Wu D.O. (2025). Enhancing routing performance through trajectory planning with DRL in UAV-aided VANETs. IEEE Trans. Mach. Learn. Commun. Netw..

[26] Kong X., Ni C., Duan G., Shen G., Yang Y., Das S.K. (2024). Energy consumption optimization of UAV-assisted traffic monitoring scheme with tiny reinforcement learning. IEEE Internet Things J..

[27] Omoniwa B., Galkin B., Dusparic I. (2023). Communication-enabled deep reinforcement learning to optimise energy-efficiency in UAV-assisted networks. Veh. Commun..

[28] Zhang M., Wu S., Jiao J., Zhang N., Zhang Q. (2022). Energy-and cost-efficient transmission strategy for UAV trajectory tracking control: A deep reinforcement learning approach. IEEE Internet Things J..

[29] Shi J., Zhou S., Man X., Li C., Guo F. (2025). Trajectory planning and tracking for UAVs with deep reinforcement learning and adaptive nonlinear MPC. Expert Syst. Appl..

[30] Ribeiro P., Coelho A., Campos R. On the energy consumption of rotary-wing and fixed-wing UAVs in flying networks. Proceedings of the 2025 20th Wireless On-Demand Network Systems and Services Conference (WONS).

[31] (2010). IEEE Standard for Information Technology—Local and Metropolitan Area Networks—Specific Requirements—Part 11: Wireless LAN Medium Access Control (MAC) and Physical Layer (PHY) Specifications Amendment 6: Wireless Access in Vehicular Environments.

[32] Ribeiro P., Coelho A., Campos R. (2024). SUPPLY: Sustainable multi-UAV performance-aware placement algorithm for flying networks. IEEE Access.

